# LncRNA LINC00460 facilitates the proliferation and metastasis of renal cell carcinoma via PI3K/AKT signaling pathway

**DOI:** 10.7150/jca.73758

**Published:** 2022-07-04

**Authors:** Feng-Juan Zhou, Sen Meng, Xiao-Feng Wu, Ping-Fu Hou, Min-Le Li, Su-Fang Chu, Jin Bai, Jun-Nian Zheng

**Affiliations:** 1 The Fourth Clinical College of Nanjing Medical University, Nanjing 211100, Jiangsu Province, China.; 2 Cancer Institute, Xuzhou Medical University, Xuzhou 221002, Jiangsu Province, China.; 3 Center of Clinical Oncology, the Affiliated Hospital of Xuzhou Medical University, Xuzhou 221002, Jiangsu Province, China.; 4 Department of Radiotherapy, the Second Affiliated Hospital of Xuzhou Medical University, Xuzhou 221002, Jiangsu Province, China.; 5 Hepatobiliary Center, The First Affiliated Hospital of Nanjing Medical University; Key Laboratory of Liver Transplantation, Chinese Academy of Medical Sciences; NHC Key Laboratory of Living Donor Liver Transplantation (Nanjing Medical University), Nanjing 210029, Jiangsu Province, China.

**Keywords:** RCC, LINC00460, proliferation, metastasis, PI3K/AKT pathway

## Abstract

Renal cell carcinoma (RCC) is one of the most prevalent cancers diseases in the worldwide. Long noncoding RNAs (LncRNAs) have been indicated as a mediator acted in tumorigenesis of RCC. LINC00460 has been reported to participate in many kinds of malignancies and promotes cancer progressions. However, the mechanism of LINC00460 on RCC is yet to be investigated. This study aimed to explore the potential function and regulation mechanism of LINC00460 in RCC. We analysed the LINC00460 expression and the prognosis in RCC patients using Gene Expression Profiling Interactive Analysis (GEPIA) and The Cancer Genome Atlas (TCGA) databases. LINC00460 level in normal renal cell line and RCC cell lines were examined by qRT-PCR. We study the effects of LINC00460 on proliferation, migration, invasion, apoptosis in RCC cells lines using a series of *in vivo* and *in vitro* experiments. RNA sequencing (RNA-seq) analysis was applied to searching potential LINC00460 related signal pathway in RCC. We identified the significant up-regulated expression of LINC00460 both in RCC tissues and cell. RCC patients with elevated LINC00460 expression have shorter survival. Up-expression of LINC00460 promoted cell proliferation, invasion and migration, meanwhile down-regulation of LINC00460 exerted inhibitory effect on these activities. We crucially identified that LNC00460 promotes development of RCC by influencing the PI3K/AKT pathway. Knockdown of LNC00460 decreased the phosphorylation of AKT and mTOR. The key finding of our study showed that LINC00460 functions as an oncogene in RCC pathogenesis by mediating the PI3K/AKT.

## Introduction

Renal cell carcinoma (RCC) is a typical malignant carcinoma originating from the renal tubules. The morbidity of RCC has been increased over the years, only next to prostate cancer and bladder cancer of adult cancer diseases 1. When patients in the early phase of kidney cancer, the asymptomatic and covert symptoms always caused the awareness less of cancer screening, which made the patients often missed the diagnosis and treatment correspondingly. When the patients were diagnosed, they were already in the advanced clinical stage with local progression occurred 2. Radical surgery is still a mainstay of treatment for patients in early-stage RCC so far 3. The prognosis of RCC patients with advanced stage remains poor although progress in treatment has occurred through clinical development, and the targeted therapy effect is still limited due to drug resistance and adverse reactions 4, 5. Therefore, unraveling the molecular mechanisms of RCC for screening novel biomarkers becomes an immediate concern.

Long noncoding RNAs (LncRNAs) have been considered as transcriptional, post-transcriptional or post-translational levels regulators of gene expression 6, LncRNAs with more than 200 nucleotides and no protein-coding capacity, participate in lots of physiological and pathological bio-behaviors 7, 8, complicating the gene regulation networks, such as gene transcription regulation, RNA processing, chromatin modification, especially in tumorigenic biological activities 9. Accumulating evidences show that LncRNAs can caused occurrence of tumor and work as oncogenes 10-12. Exactly as previous studies, LncRNA MALAT1, LncRNA XIST, LncRNA HOTAIR or LncRNA TUG1 facilitate tumor cell growth, migration or invasion in multiple types of tumors such as cervical cancer, triple-negative breast cancer, gastric cancer, multiple myeloma, pancreatic cancer, bladder cancer and thyroid cancer 13-17.

Long intergenic noncoding RNA 460 (LINC00460) is a novel carcinogenic LncRNA related in the development of different kinds of human malignancies, including lung cancer, thyroid cancer, nasopharyngeal carcinoma 10, 18, 19. When we found that high expression of LINC00460 was associated with poor prognosis in RCC patients through TCGA database analysis, which caught our attention to study the biofunction of LINC00460 in RCC. Sadly, the more specific mechanism of the LINC00460 function in RCC is still unclear.

The phosphatidylinositol 3-kinase (PI3K)/AKT/mammalian target of rapamycin (mTOR) signaling pathway aberrant activation regulated cellular processes including proliferation, metabolism, apoptosis and metastasis in many cancer types 20-22. Study reported that PI3K/AKT/mTOR signaling pathway promote human esophageal cancer tumorigenesis and metastasis, induce potential cell apoptosis of the esophageal squamous cell carcinoma 23. This remarkable pathway is regulated by a number of upstream signaling and its cascades downstream signaling pathways collaborating with various compensatory effectors 24.

In this study, we analyzed data from the TCGA database using the online bioinformatics tool GEPIA and found the increased LINC00460 expression level in RCC tissues predicted poor survival of RCC patients. We evaluated the level of LINC00460 in RCC cells and its tumorigenesis on RCC cell vitality, migration, invasion and apoptosis *in vitro* and *in vivo*. RNA sequencing (RNA-seq) analysis proved that LINC00460 knockdown could mainly affect the genes involved in proliferation and apoptosis. In the viewpoint of the mechanism, our results indicated that LINC00460 mediates PI3K/AKT signaling to promote the progression of RCC cells. Thus, this study presented a perspective understanding of RCC pathogenesis. We deeply revealed the mechanism of LINC00460 function in RCC.

## Materials and Methods

### Bioinformatical analyses

The Gene Expression Profiling Interactive Analysis (GEPIA) and The Cancer Genome Atlas (TCGA) databases were used to analyzed LINC00460 expression in RCC and normal tissue. Clinicopathological features including overall survival (OS) and disease-free survival (DFS) was evaluated linked to RCC patients LINC00460 expression.

### Cell Culture

Human embryo kidney epithelial cell line (HK-2) and renal cell carcinoma cell lines were purchased from cell bank of Chinese Academy of Sciences. ACHN, 786-O, OSRC-2, Ketr-3, and HK-2 were correspondingly cultured in RMPI-1640 or DMEM Medium, supplemented with 10% FBS, 100 U/mL each penicillin and streptomycin separately. Cell incubated condition is 37 °C humidified with 5% CO2.

### Cell Transfection

For over-expressed LINC00460, sequences were constructed into pCDH-CMV-MCS-EF1-GreenPuro lentivirus vector (GenePharma Suzhou, China). shLINC00460#1 (5'-GCTAAGACCTAATAGCCAATA-3'), shLINC00460#2 (5'-ACCTTGGTCAAACGTTTAACC-3'), as well as the negative control shCtrl (5'-GTTCTCCGAACGTGTACGT-3') were constructed into pLKO.1 (GenePharma Suzhou, China) for knockdown of LINC00460. psPAX2, pMD2.G and pLKO-shRNA were co-transfected into HEK-293T cells for the lentiviruses until 48h later. Stable knockdown ACHN and 786-O cell lines with a treatment of 5 μg/mL puromycin for 1 week were obtained after lentiviruses infected.

### RNA isolate, RT-PCR and qPCR

We prepared total RNA by using TRIzol Reagent (Invitrogen), synthesized cDNA with HiScript Q RT SuperMix for qPCR Kit (Vazyme Biotech). Quantitative realtime PCR was performed on ABI-7500 using UltraSYBR Mixture Kit (CWBIO Biotech). The primers using for qPCR analysis were listed as followed: LINC00460 Forward: ACGCAGTGGATGAGAACGAA, LINC00460 Reverse: GGGGTGACTTCAGAATGCGT, 18S rRNA Forward: GTAACCCGTTGAACCCCATT, 18S rRNA Reverse: CCATCCAATCGGTAGTAGCG.

### Cell Counting Kit 8 (CCK-8)

CCK-8 (Beyotime, China) was used to measure cell proliferative capacity. 96-well plates planted with transfected cells (5×10^3^ cells/group) were incubated at 37 °C with 5% CO_2_ for 24 h, 48 h, 72 h. 10 µl of CCK-8 solution was added to each cell well at each time point and incubate for 2h subsequently. The absorbance at 450 nm of each group was tested by the microplate reader.

### Transwell Assays

Firstly, ACHN or 786-O cells were planted into the upper wells of chambers (BD Biosciences, USA) coated with or without Matrigel (BD Biosciences), then, cultured in a 200 μL FBS-free RMPI-1640 or DMEM medium. The lower wells of the chambers containing 390 μL culture medium and 10 μL FBS. 24 hours later, removed the medium of the upper wells, and fixed cells with 100% methanol and then stained with 0.1% crystal violet for 15 min. The stained cells were photographed using an Olympus microscope.

### Wound Healing Assays

Gaps in seeded ACHN or 786-O cells were created by a sterilized pipette tip. After washing out the debris or the detached cells by PBS, the cells were cultured in RMPI-1640 or DMEM for another 24 hours. The calculation of wound healing width was using ImageJ Analysis Software.

### Flow cytometry

Flow cytometry (BD, UA) was used to examine cell apoptosis. No stimulation was given to RCC cells to produce apoptosis. EDTA-free trypsin digested 786-O and ACHN cells. Then, the cells were suspended in 1× binding buffer at 3×10^6^/mL. Subsequently, 100 μL of cells were moderately filled with each of 5 μL of APC and PI, followed by incubation with no light for 5 min at room temperature. Cell apoptosis was analyzed using the flow cytometer. The number of Annexin-APC positive cells represented cell apoptosis rate analyzed by FlowJo v10.6.2 software.

### Western Blot

The total content of cellular protein harvested from RCC cells were extracted using RIPA lysis buffer (Keygen, Nanjing, China) and the protein amounts was determined using a BCA kit (Keygen, Nanjing, China). SDS-PAGE separated same volumes proteins of each lane, then blotted onto PVDF membranes. Primary antibodies were incubated overnight at 4 °C, and HRP-labeled secondary antibody (ABclonal, 1:5,000) 2 hours at RT after blocked with 5% BSA. Signal of immunoreactivities were photographed by ECL reagent (NCM Bio, China) on Tanon 5200 automatic chemiluminescence imaging system (Tanon, China). Antibody listed as follow: Anti-E-cadherin (BD Biosciences, 610181), Anti-Vimentin (Proteintech, 10366-1-AP), Anti-N-cadherin (BD Biosciences, 610920), Anti-PI3k (Proteintech, 67121-1-Ig), Anti-AKT (Proteintech, 101762-2-AP), Anti-p-AKT (Proteintech, 66444-1-Ig), Anti-p-mTOR (Cell Signaling Technology, 5536S), Anti-Bcl-2 (Cell Signaling Technology, 155071S), Anti-Cleved-Caspaes 9 (Cell Signaling Technology, 20750S), Anti-p53 (Proteintech, 10442-1-AP), and we took GAPDH(Santa Cruz, sc-32233) as control.

### Animal Works

We used BALB/c mice (Vital River Laboratory Animal Technology. China) for research. We subcutaneously injected 786-O cells (5×10^6^) of each group (shCtrl and shLINC00460) into the two side of mice. When tumors were visible, we recorded xenograft tumors volume (V) every 3 days by metering the long axis (L) and the short axis (W), and calculated the tumors' growth rate with the equation: V = (L × W2)/2. By day 27, the xenograft tumor tissues were harvested, weighed, photographed and subjected to subsequent analysis.

Meanwhile, shCtrl and shLINC00460 786-O-Luc cells (3 × 10^6^) were injected intravenously via the mice tail vein. Bioluminescence images were filmed after 6 weeks (Night OWL II LB983; Berthold Technologies). Animal Care and Use Committee and Ethics Committee of Xuzhou Medical University approved all animal experiments.

### Statistical Analysis

All collected data were analyzed using Statistical Product and Service Solutions (SPSS 23.0) (IBM, USA) and presented in form of mean ± standard deviation. Student's t-test were used to tested the differences between two groups. Comparison among three groups was analyzed using One-way ANOVA test. *p*<0.05 suggested statistically significant differences.

## Results

### LINC00460 expression was elevated and closely linked to poor prognosis in RCC

**Figure 1:**
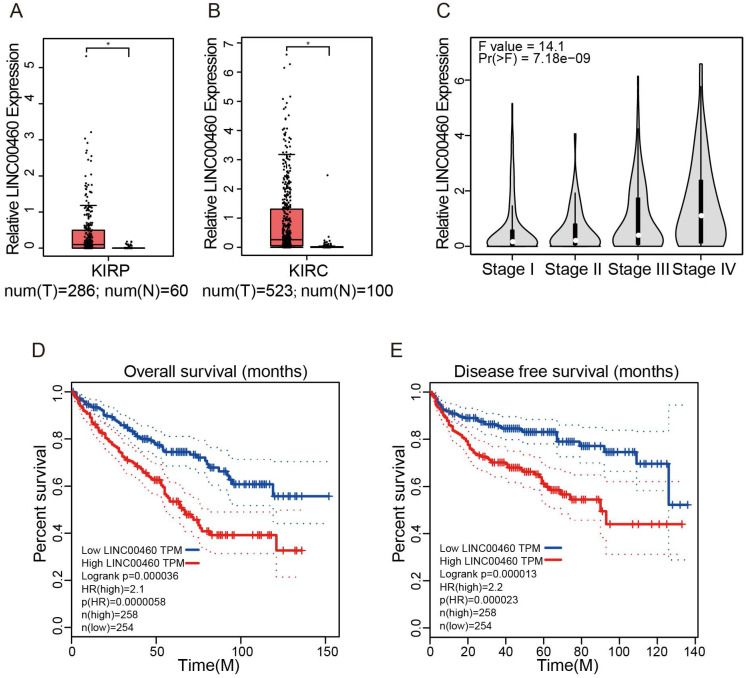
** Expression of LINC00460 in RCC tissues based on the data from the GEPIA and TCGA. (A, B)** Expression level of LINC00460 in KIRP tissues (n=286) and normal tissues (n=60), KIRC tissues (n=523) and normal tissues (n=100), analyzed in TGCA database (fold change>2.0, * *p*<0.05). **(C)** LINC00460 was gradually elevated with advanced staging in RCC. **(D, E)** OS and DFS rate of RCC patients with lowly or highly LINC00460 analyzed using the Kaplan-Meier analyses and log-rank test.

GEPIA database with TCGA datasets were used to obtain LINC00460 expression in RCC tumor tissues paired with normal tissues and the survival of RCC patients. GEPIA with TCGA database revealed that LINC00460 was higher level both in Kidney renal clear cell carcinoma (KIRP) and Kidney renal papillary cell carcinoma (KIRP) (Fig. 1A, 1B). In addition, clinicopathological staging are important prognostic factors for RCC patients, we investigated the LINC00460 expression patterns in different clinical pathological grade of RCC in GEPIA database, we identified a gradual increase in the expression of LINC00460 with the RCC pathological progression (Fig. 1C). Furthermore, RCC patients with high expression of LINC00460 exhibited a shorter OS and DFS than those with low expression of LINC00460 (Fig. 1D, 1E). The results indicated that aberrant expression of LINC00460 might be strongly associated with poor prognosis in RCC.

### LINC00460 was elevated in RCC cell Lines and promoted cell proliferation

**Figure 2:**
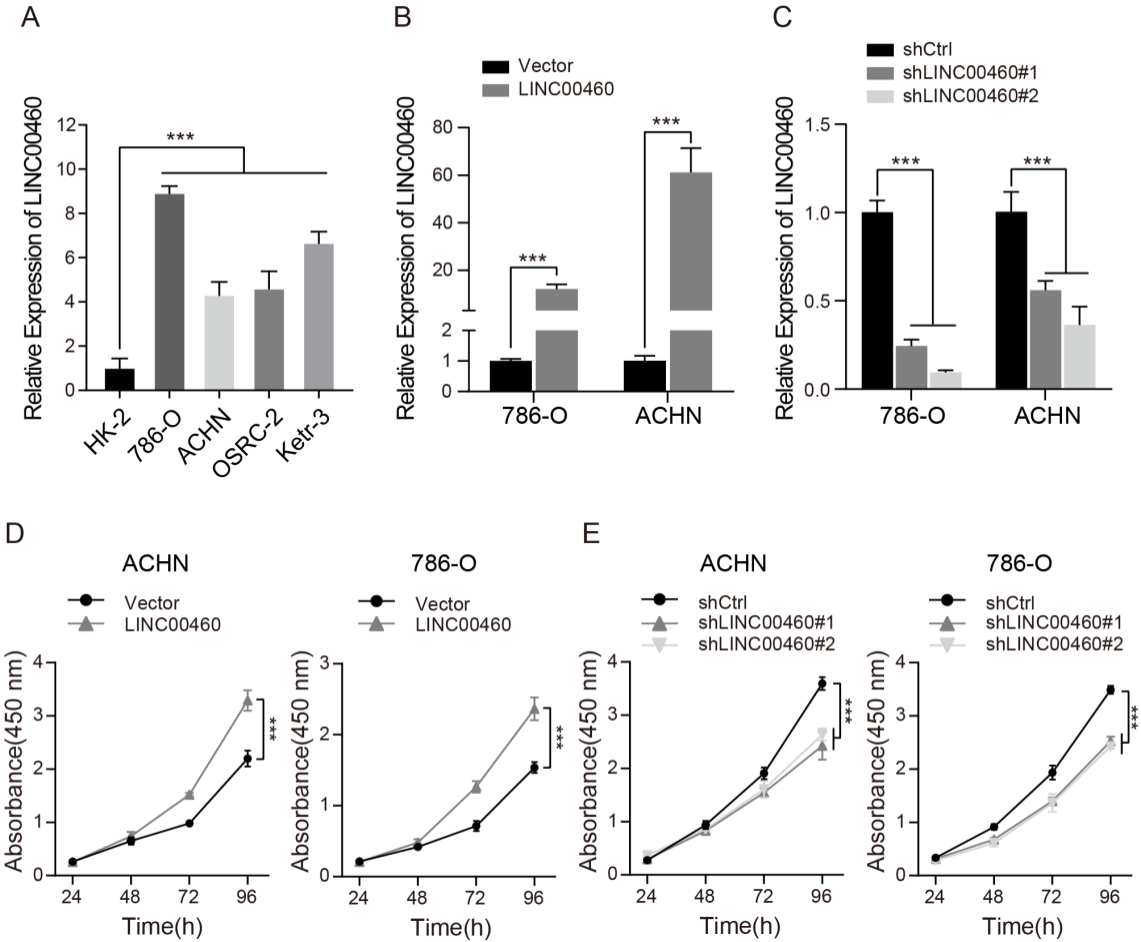
** LINC00460 expression level in RCC cell lines and effects on cell proliferation. (A)** Expression patterns of LINC00460 in RCC cell lines and normal renal cell lines detected by qRT-PCR (*** *p*<0.001). **(B, C)** Expression patterns of LINC00460 in RCC cells treated with over-expressed or silenced LINC00460 detected by qRT-PCR (*** *p*<0.001). **(D, E)** CCK-8 assay of ACHN and 786-O cells transfected with over-expressed or silenced LINC00460 (*** *p*<0.001).

We detect the expression of LINC00460 in RCC cells to explore the effect of LINC00460 on RCC progression. qRT-PCR analysis demonstrated that LINC00460 was significantly upregulated in RCC cell lines compared with normal renal cell (Fig. 2A). Subsequently, qRT-PCR was used to measure the LINC00460 expression level after different transfection applied to the cells, and the results showed that LINC00460 was significantly upregulated in cells transfected with LINC00460 overexpression vector (Fig. 2B), while LINC00460 was remarkably downregulated in cells transfected with the pLKO.1-shRNA, compared with the control vector group (Fig. 2C). Next, we tested cell proliferation by CCK-8 assay. The data of CCK-8 revealed that upregulated LINC00460 expression increased cell proliferation as relative to the control groups (Fig. 2D), while downregulated LINC00460 expression suppressed cell proliferation (Fig. 2E).

### LINC00460 facilitated RCC cell migration, invasion and induced EMT phenotype *in vitro*

**Figure 3:**
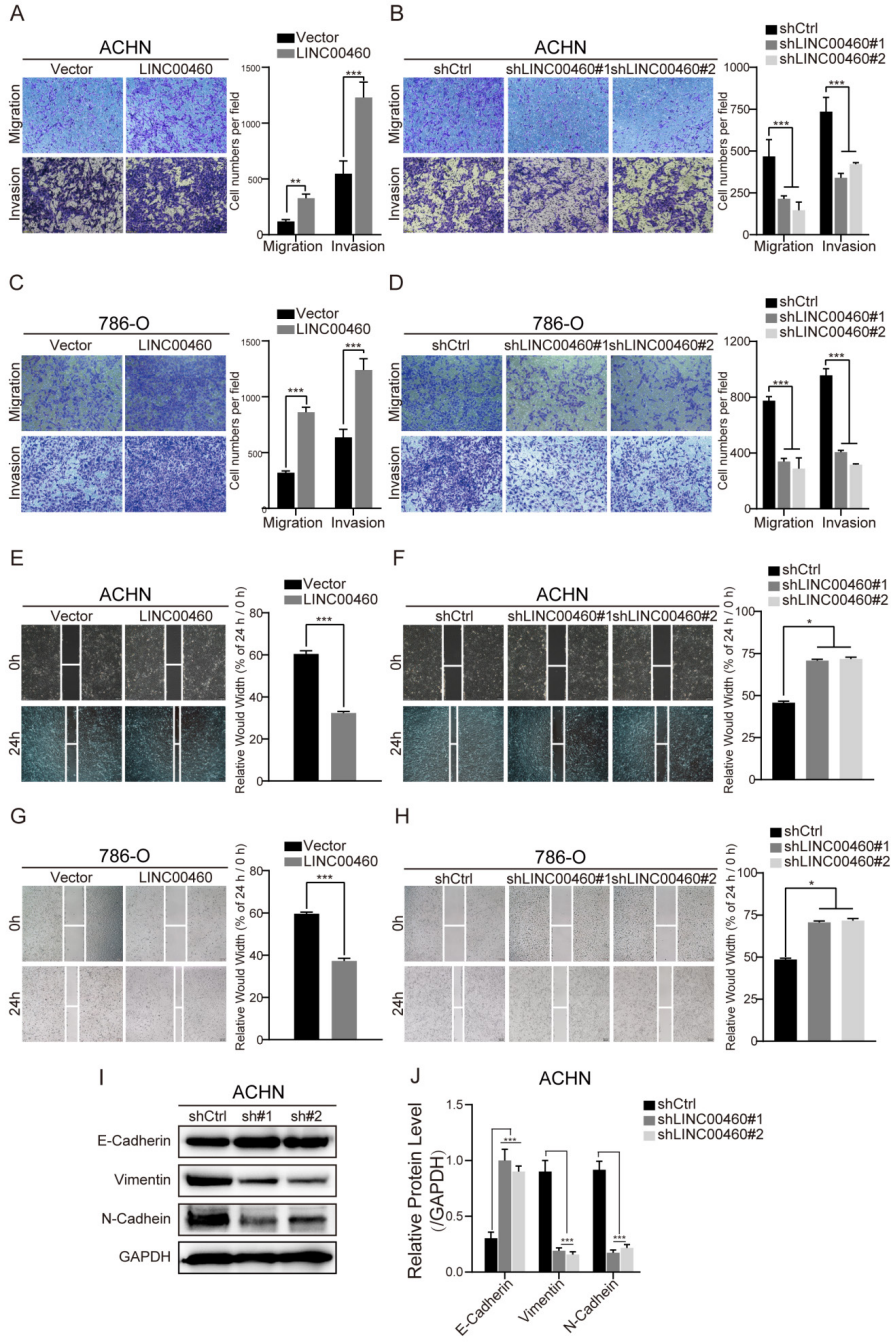
** LINC00460 facilitated RCC cells migration, invasion *in vitro*. (A, B, C, D)** The cell migration and invasion abilities of ACHN and 786-O cells transfected with overexpressed or silenced LINC00460 were determined by Transwell assays (*** *p*<0.001). **(E, F, G, H)** Wound healing assay was performed to examine the effect of LINC00460 overexpression or knockdown on ACHN and 786-O cells migration (* *p*<0.05, *** *p*<0.001). **(I, J)** EMT markers was detected by Western blot analysis when silenced LINC00460 was transfected in RCC cells. Data statistics was also shown (GAPDH as negative control, *p < 0.05, *** *p*<0.001).

In transwell assays, we showed that LINC00460 overexpression enhanced ACHN and 786-O cells migration and invasion (Fig. 3A, 3C), LINC00460 knockdown inhibited migration and invasion of ACHN and 786-O cells (Fig. 3B, 3D). We conformed the same results that LINC00460 overexpression enhanced cell migration by wound healing assays (Fig. 3E, 3G), while LINC00460 knockdown suppressed cell migration (Fig. 3F, 3H) respectively.

We detect the expression of EMT-related proteins inevitably, as the outcome obtained from Western blot analysis (Fig. 3I, 3J). Knockdown of LINC00460 increased the levels of EMT marker E-cadherin and decreased N-cadherin and Vimentin protein levels in ACHN cells. Consistent with the results, we can make the conclusion that LINC00460 could accelerate the invasion, migration and induced EMT progress of RCC cells.

### LINC00460 affected the apoptosis of RCC cells

**Figure 4:**
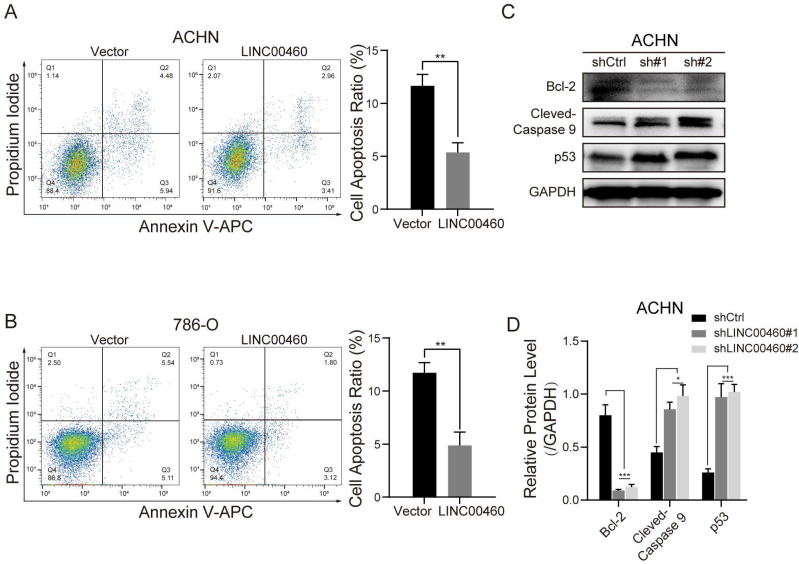
** Influence of LINC00460 on RCC cells apoptosis and EMT phenotype. (A, B)** Cell apoptosis was determined using flow cytometry analysis after LINC00460 overexpression in RCC cell lines (** *p*<0.01). **(C, D)** Cell apoptosis markers were detected by Western blot analysis when silenced LINC00460 was transfected in RCC cells. Data statistics was also shown (GAPDH as negative control, *p < 0.05, *** *p*<0.001).

Flow cytometry and Western blot assay were associated to verify whether promotion of cell malignant progress by LINC00460 was associated with cell apoptosis. Compared with shCtrl group, apoptotic cells were decreased in the LINC00460 over-expressed ACHN or 786-O cells (Fig. 4A, 4B). Anti-apoptotic protein Bcl-2 was diminished after LINC00460 knockdown, conversely, pro-apoptotic protein Cleaved Caspase-9 and p53 expression were increased in ACHN cells (Fig. 4C, 4D). All of these results proved that LINC00460 affects the malignant progression of RCC cells by affecting apoptosis.

### LINC00460 promoted RCC cell growth and metastasis *in vivo*

**Figure 5:**
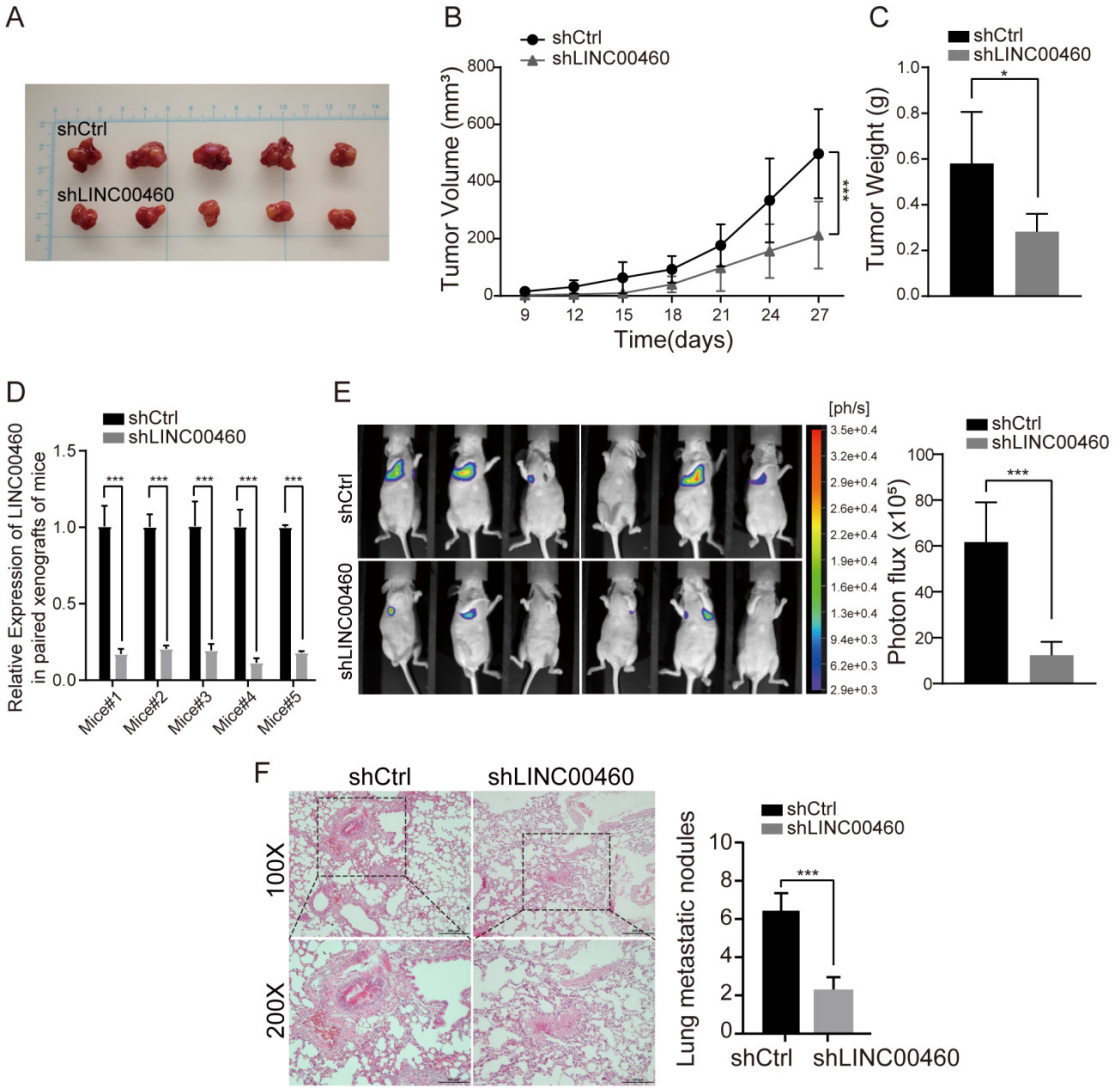
** LINC00460 promoted RCC cells growth and metastasis *in vivo*. (A)** 786-O cells were injected into nude mice after the transfection of shLINC00460 and shCtrl vector. **(B)** The growth curves of tumors from subcutaneously injected nude mice treated with shLINC00460 or shCtrl (*** *p*<0.001). **(C)** The weights of tumors from subcutaneously injected nude mice treated with shLINC00460 or shCtrl (* *p*<0.05). **(D)** The levels of LINC00460 expression in paired tumor tissues formed from subcutaneously injected nude mice treated with shLINC00460 or shCtrl, determined by qRT-PCR (*** *p*<0.001). **(E)** Representative bioluminescence images and statistical analysis of lung metastases in mice via tail vein injection of indicated cells (**p < 0.01, ***p < 0.001). **(F)** Representative H&E images and statistical analysis of resected lung xenografts with metastatic loci (*** *p* < 0.001).

For the assessment of LINC00460 influence on tumor growth *in vivo*, nude mice were injected subcutaneously with transfected 786-O cells. Knockdown of LINC00460 noticeable suppressed proliferation of tumor cells (Fig. 5A), as presented by the markedly reduced tumor volume, size and weights in the knockdown group (Fig. 5B, 5C). Furthermore, the qRT-PCR results confirmed that the significantly lower level of LINC00460 in shLINC00460-treated groups of xenograft tumor tissues compared with shCtrl groups (Fig. 5D).

To further evaluate LINC00460 knockdown could produce inhibition effect on metastasis *in vivo*, we administered tail vein injections with 786-O-Luc cells stably transduced shLINC00460 or shCtrl vector into two groups of mice each respectively. Six weeks after tail vein injections, we used bioluminescence photograph system to assess the metastatic nodules formed on the lung surfaces of mice. Group with LINC00460 knockdown cells formed a smaller number of metastatic foci in lungs than the control group (Fig. 5E, 5F). Overall, the data demonstrated that LINC00460 downregulated could inhibit RCC cell growth and metastasis *in vivo*.

### Dysregulation of LINC00460 was related to PI3K/AKT pathway

**Figure 6:**
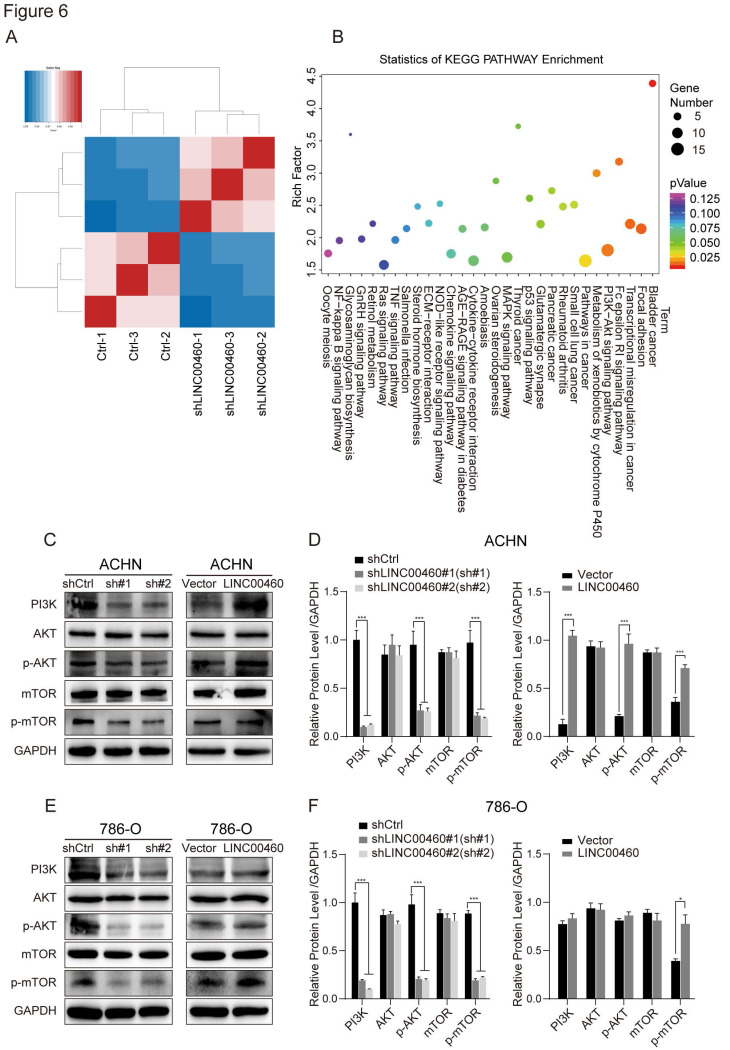
** Effect of LINC00460 knockdown on activation of PI3K/AKT pathway in RCC cell lines.** (A) Hierarchically clustered heatmap of the upregulated and downregulated genes in 786-O cells after shLINC00460 and shCtrl transfections. (B) Pathway classification of differentially expressed genes (DEGs). Bubble plots represented the number of DEGs, x axis represented rich factor, y axis represented the functional classification of KEGG. (C, D, E, F) The Western blot assay was used to detect PI3K, AKT, p-AKT, mTOR and p-mTOR expression in cells transfection with shLINC00460 or overexpressed LINC00460 as relative to the control groups. (GAPDH as negative control, *p < 0.05, *** *p*<0.001).

To explore more particular details about the LINC00460 involved pathway in RCC, RNA-seq was processed after LINC00460 knockdown in 786-O cells (Fig. 6A). To get further investigation of LINC00460 worked in RCC, Kyoto encyclopedia of genes and genomes (KEGG) pathway enrichment analysis was conducted for looking the target genes of LINC00460 (>1.5 fold change). KEGG pathway clustering indicated that the significantly over-represented biological pathways mapping in cell motility, cell growth and death, cell repair and replication. Markedly, the dysregulated key genes that were related to the PI3K/AKT pathways (Fig. 6B).

PI3K/AKT pathway was involved in different cancer cell behaviors, especially cell proliferation and apoptosis 25. Thus, we investigated whether LINC00460 control cancer cell progress by the PI3K/AKT pathway. The Western blot assay revealed a reduction of PI3K, p-AKT and p-mTOR expression in ANCH and 786-O cells transfected with shLINC00460 as relative to the control groups. Meanwhile, the expression of PI3K, p-AKT and p-mTOR were increased in cells transfected with overexpressed LINC00460 compared with the control groups (Fig. 6C-F). These results suggested that dysregulation of LINC00460 could regulate the PI3K/AKT pathway.

## Discussion

Renal cell carcinoma (RCC) is a heterogenous cancer consisting of various different subtypes 26, and considered as a stepwise process involving the accumulation of multiple genetic and epigenetic alterations 27. The most critical biological characteristics of RCC are uncontrolled cellular proliferation, abnormal apoptosis and metastasis, which are the leading cause of mortality 28. Unfortunately, many patients have been in advanced RCC stage when diagnosed due to no effective diagnosis biomarker and a poor understanding of the mechanism.

To date, LncRNAs have been known as active biological marker in cancer diagnosis rather than transcriptional noise 29. They were proved to play pivotal roles in carcinogenesis via regulating various important cellular biological behavior, including proliferation, apoptosis, angiogenesis, invasion and metastasis 30. In cervical cancer, upregulation of LncRNA ZEB1-AS1 enhances cell epithelial to mesenchymal transition by elevating ZEB1 level 31. LncRNA UCA1 is increased in thyroid cancer and represses cell proliferation and cell invasion by interacting with miR-204/IGFBP5 32. LncRNA CA3-AS1 restraint of colorectal cancer cell vital, invasion by miR-93/PTEN axis 33. The downregulation of LINC00152 suppresses the development of gastric cancer through controlling miR-193b-3p 34. To our knowledge, LINC00460 has been reported to participate in many kinds of malignancies and promotes cancer progressions.

In the present research, LINC00460 have been proved overexpressed in RCC according to the TCGA analysis of clinical specimens, higher LINC00460 expression level was correlated with poor OS and DFS and advanced clinicopathological staging in RCC patients, which is key finding of prognostic significance of LINC00460 for RCC patients. Our work is the initial presentation of LINC00460 biologic function, correlated with progress of RCC cells. A series of assays *In vitro* and *in vivo* revealed that LINC00460 over-expression enhanced cell proliferation, migration, and invasion, while its down-regulation suppressed tumor growth and reduced cell migration and invasion. Flow cytometry assays showed that overexpressed LINC00460 inhibited apoptosis in RCC cells. To routinely and precisely unravel the pathways that were related to the function of LINC00460 in tumorigenesis of RCC, RNA-seq and date analysis was conducted, and PI3K/AKT pathway was found to be regulated by LINC00460. Then we confirmed this finding via Western blot assays. These findings indicate that LINC00460 plays an oncogenic role in RCC development and is potential prognostic marker for RCC patients.

Emerging studies have identified that the PI3K/AKT signaling pathway influences various pathophysiological progress that associated with cancer phenotypes, such as cell apoptosis and cell proliferation 35-40. PI3K activation phosphorylates AKT and active AKT can lead to a number of downstream effects including the activation of mTOR, also in the form of phosphorylates mTOR, which in turn directly impacts cell growth and survival 41-43. The crucial influence of the activation of AKT is the increasing cell viability 44, and involved in cellular tumorigenicity behaviors such as proliferation, metastasis, invasion, migration, and angiogenesis 45, 46. Therefore, in accordance with our RNA-seq data analysis, we found that the knockdown of LINC00460 downregulated the expression of p-mTOR, p-AKT and PI3K, confirmed our hypothesis that LINC00460 might act as one upstream of PI3K/AKT pathway to control RCC progression. Furthermore, our findings displayed in Western blot assay indicating that LINC00460 could promote tumor migration and invasion via EMT.

## Conclusions

In conclusion, our findings illustrated that LINC00460 expression was strongly upregulated in RCC tissues and cells, consequently, its higher expression might be associated with poor clinical prognosis in RCC patients, which enables it as an attractive prognostic factor for RCC. LINC00460 influenced tumor growth, metastasis and apoptosis *in vitro* and *in vivo*. In the aspect of signaling pathway, LINV00460 activating the PI3K/AKT pathway. Our findings might display a novel insight into the mechanism of LINC00460 in RCC. The mechanism about LINC00406 how to regulate PI3K/AKT signaling pathway are still needed our further study to verify. Further clinical experiments are still needed to verify.

## Abbreviations

RCC: Renal cell carcinoma
KIRC: Kidney renal clear cell carcinoma
KIRP: Kidney renal papillary cell carcinoma
KICH: Kidney Chromophobe
LincRNA: Long intergenic noncoding RNA
GEPIA: Gene Expression Profiling Interactive Analysis
TCGA: The Cancer Genome Atlas
qRT-PCR: Quantitative real-time polymerase chain reaction
RNA-seq: RNA sequencing
OS: Overall survival
DFS: Disease-free survival
LncRNAs: Long noncoding RNAs
LINC00460: Long intergenic noncoding RNA 460
PI3K: The phosphatidylinositol 3-kinase
mTOR: Mammalian target of rapamycin
p-AKT: Phosphorylated AKT
p-mTOR: Phosphorylated mTOR
EMT: Epithelial-mesenchymal transition
PI: Propidium Iodide
